# Genome characterization of Turkey Rotavirus G strains from the United States identifies potential recombination events with human Rotavirus B strains

**DOI:** 10.1099/jgv.0.000963

**Published:** 2017-11-23

**Authors:** Fangzhou Chen, Todd P. Knutson, Robert E. Porter, Max Ciarlet, Sunil Kumar Mor, Douglas G. Marthaler

**Affiliations:** ^1^​ Department of Veterinary Population Medicine, College of Veterinary Medicine, University of Minnesota, St. Paul, MN 55108, USA; ^2^​ State Key Laboratory of Agricultural Microbiology, College of Veterinary Medicine, Huazhong Agricultural University, Wuhan 430070, PR China; ^3^​ Vaccines Clinical Research and Development, GlaxoSmithKline Vaccines, Cambridge, MA 02139, USA; ^4^​ Department of Diagnostic Medicine and Pathobiology, College of Veterinary Medicine, Manhattan, KS 66506, USA

**Keywords:** Rotavirus G, phylogenetic

## Abstract

Rotavirus G (RVG) strains have been detected in a variety of avian species, but RVG genomes have been published from only a single pigeon and two chicken strains. Two turkey RVG strains were identified and characterized, one in a hatchery with no reported health issues and the other in a hatchery with high embryo/poult mortality. The two turkey RVG strains shared only an 85.3 % nucleotide sequence identity in the VP7 gene while the other genes possessed high nucleotide identity among them (96.3–99.9 %). Low nucleotide percentage identities (31.6–87.3 %) occurred among the pigeon and chicken RVG strains. Interestingly, potential recombination events were detected between our RVG strains and a human RVB strain, in the VP6 and NSP3 segments. The epidemiology of RVG in avian flocks and the pathogenicity of the two different RVG strains should be further investigated to understand the ecology and impact of RVG in commercial poultry flocks.

Rotaviruses (RVs) are causative agents of acute viral gastroenteritis and diarrhoea in multiple animal species [1]. Belonging to the family *Reoviridae*, RVs consist of a non-enveloped virion encasing 11 double-stranded RNA segments, which encode for 5–6 non-structural proteins (NSP1-NSP5/NSP6) and 6 viral proteins (VP1-4, VP6 and VP7) [2]. The outer capsid of the virion is composed of the VP7 and VP4 proteins while the middle and inner layers of the capsid are formed by VP6 and VP2 proteins, respectively. The VP1 protein is an RNA-dependent RNA polymerase that coordinates rotavirus RNA packing and genome replication [3]. The VP3 protein is a multi-functional protein associated with guanylyltransferase [4] and methyltransferase [5] activities. The NSP1 protein is an important innate host immune response regulator that can inhibit expression of type I interferon [6] and counteract NFκB [7]. The NSP2 and NSP5 proteins mediate the assembly of viroplasm [8, 9]. The NSP3 protein can regulate viral mRNA translation by simultaneous interaction with eukaryotic translation initiation factor eIF4G and the mRNA 3′ end [10]. The NSP4 protein is a multi-functional viral enterotoxin [11].

Currently, RV species are classified by the inner capsid protein VP6, and strains within the same rotavirus species share >60 % nucleotide identity [12]. Eight official RV species have been identified (RVA-RVH), and two tentative new RV species (RVI and RVJ) were identified in dogs [13] and bats [14], respectively. Due to their high prevalence and pathogenicity, RVA is well characterized and has been identified in both mammalian and avian species [15, 16]. RVB, RVC, RVE and RVH have detected in various mammalian species, while RVD, RVF and RVG have been exclusively identified in different avian species [1]. Rotavirus-like viruses were first identified in intestinal contents specimens from turkey flocks by immune electron microscopy and genome electropherotyping methods in the USA [17]. RVD was first identified in chicken faeces [18–20], and later identified in Europe and Asia [21]. RVF was identified in turkey faeces [17] and was detected in broiler chicks with runting and stunting syndrome [22]. RVG was first identified in a chicken from Northern Ireland [18] and later detected in turkeys [23]. Subsequently, RVG has been identified in Brazil, Germany, Italy, the Netherlands and South Africa [24–26]. RVG was found in broiler chicks with runting and stunting syndrome [22], but its role in the diseases remains unclear.

For RVA, a sequence-based classification system was proposed in 2008 [27]. Subsequently, the Rotavirus Classification Working Group (RCWG) was involved in RVA classification [28]. To understand evolution and reassortment between host species, hundreds of complete genome sequences of different RV species have been reported, but turkey RVG sequences are lacking. Since RVG has not been isolated in cell culture, molecular epidemiology is the main approach to characterizing RVG strains to understand the genetic diversity and evolution compared to RVA. In the present study, we identified and characterized the genome sequences of two US turkey RVG strains identified from a hatchery with no reported signs of disease (RVG/turkey-wt/Minnesota-1/USA/2016) and a hatchery with high embryo/poult mortality (RVG/turkey-wt/Minnesota-2/USA/2016).

The intestinal caecum samples from 3-day-old poults from two Minnesota hatcheries (MN-1=hatchery with no disease and MN-2=hatchery with high embryo/poult mortality and lesions of white, caseous exudate within dilated caeca) were submitted to the Veterinary Diagnostic Laboratory of the University of Minnesota. The caeca of two poults from each hatchery were pooled and tested for routine bacterial culture and conventional multiplex PCR for turkey enteric viral pathogens, including RVA, astrovirus type 2 and reovirus. Both pools were negative for RVA, astrovirus and reovirus by RT-PCR. The sample from MN-1 was negative on bacterial culture while MN-2 was positive for Salmonella. The two pools were individually analysed by Next Generation Sequencing (NGS) on Illumina MiSeq (2×250 bases). Rotavirus G was the only viral pathogen identified in the samples, using a metagenomics pipeline [29]. The pool from MN-1 contained 281 073 (12.6 %; 281 073/2 233 562) RVG reads while the pool from MN-2 contained 10 393 (0.3 %; 10 393/3 080 286) RVG reads. The RVG reads were *de novo* assembled using bowtie2 V2.2.4 [30], resulting in the 11 segments of RVG. The genome sequences of RVG/turkey-wt/Minnesota-1/USA/2016 and RVG/turkey-wt/Minnesota-2/USA/2016 were deposited in GenBank under the accession nos KY689676–KY689686 and MF120214–MF120224, respectively.

Blast was performed on the RVG gene segments, which indicated high nucleotide identity to human RVB VP6 and NSP3 gene segments. Thus, RDP4 [31] was used to investigate potential recombination events between turkey RVG strains and human RVB strains. The sub-programmes GENECONV, MaxChi and Chimaera in RDP4 detected potential recombination in the VP6 gene segment, where the turkey RVG strains harboured a similar region in the 110–230 nucleotides to the human RVB/Human-wt/BAN/Bang117/2002 (GenBank accession no. GU391305.1) Fig. 1(a), which was visualized using a similarity plot with a two-parameter (Kimura) distance model and a sliding window of 60 base pairs and step size of 10 base pairs. With RDP4, GENECONV, BootScan, SiScan and TOPAL sub-programmes detected potential recombination between the turkey RVG strains, which harboured a similar region in the 130–270 nucleotides to the human RVB/Human-wt/JAP/MMR-B1/2007 (GenBank accession no. GU370059.1) Fig. 1(b). While recombination within a RV species is rare, recombination with different RV species is unlikely to occur [32]. This identification of potential recombination could be an artefact related to protein function, which is necessary for viral persistence. Blastp analysis of the 11 RVG proteins indicated low amino acid identity (<60 %) to RVB proteins, supporting an evolutionary or biological relationship between RVG and RVB.

**Fig. 1.**
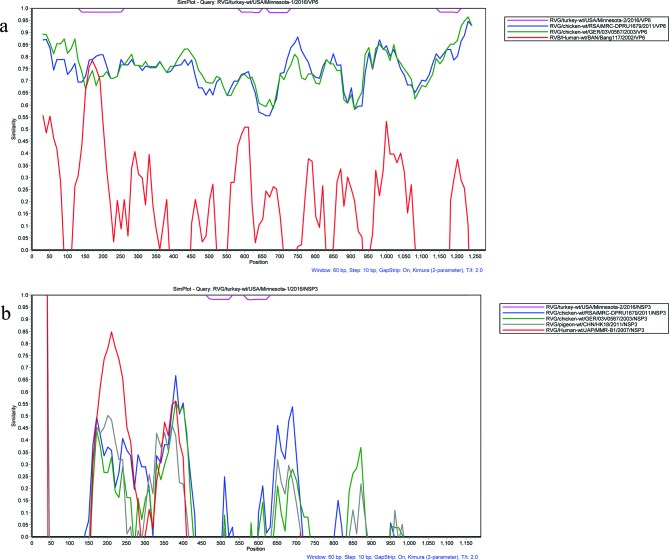
Similarity plot of different segments of avian RVG strains and human RVB strain. (a) Similarity plot of the complete VP6 gene of RVG strains and human RVB strain: RVB/Human-wt/BAN/Bang117/2002. (b) Similarity plot of the complete NSP3 gene of RVG strains and human RVB strain: RVB/Human-wt/JAP/MMR-B1/2007. The study strain, RVG/turkey-wt/USA/Minnesota-1/2016/VP6, was set as query strain in both analyses. The vertical and horizontal axes indicate the nucleotide sequence identities and nucleotide position (bp) in the graph, respectively.

The RVG gene segments were aligned with publically available RVG sequences in GenBank, and nucleotide and amino acid sequences identities were calculated with Lasergene package MegAlign software v7.1.0 (DNASTAR, Inc., Madison, WI, USA). The RVG VP6 sequences from MN-1 and MN-2 share 66.2–79.1 % of their nucleotide identities with the reported three RVG strains, confirming the identification of turkey RVG [12]. The length of the open reading frame (ORF) along with nucleotide and amino acid identities of the MN-1 gene segments were compared to the corresponding segments of RVG/turkey-wt/Minnesota-2/USA/2016, chicken and pigeon RVG strains (Table 1). When comparing MN-1 and MN-2, the RVG ORFs of VP7, VP4, VP6, VP1-VP3, NSP1, NSP2, NSP4 and NSP5 were the same length, except for the NSP3 protein, which was 63aa longer (Table 1).

**Table 1.** Nucleotide and amino acid sequence identities (%) and lengths of coding regions of RVG/turkey-wt/USA/Minnesota-1/2016 compared to the four other RVG strains Length of the nucleotide and/or amino acid coding region is in parentheses.

Turkey-wt/USA/ Minnesota-1/2016	Turkey-wt/USA/Minnesota-2/2016		Chicken-wt/RSA/MRC-DPRU1679/2011		Chicken-wt/GER/03V0567/2003		Pigeon-wt/CHN/HK18/2011	
	Nucleotide	Amino acid	Nucleotide	Amino acid	Nucleotide	Amino acid	Nucleotide	Amino acid
VP7 (750, 249)	85.3 % (750)	88.4 % (249)	61.6 % (744)	57.0 % (247)	57.0 % (744)	46.2 % (247)	53.9 % (744)	47.8 % (247)
VP4 (2367, 788)	97.9 % (2367)	99.1 % (788)	48.0 % (2340)	36.8 % (779)	48.4 % (2319)	37.9 % (772)	48.6 % (2268)	36.3 % (755)
VP6 (1176, 391)	99.6 % (1167)	100 % (391)	78.7 % (1176)	25.7 % (391)	79.1 % (1176)	25.1 % (391)	66.2 % (1176)	45.7 % (391)
VP1 (3483, 1160)	99.9 % (3482)	99.8 % (1160)	82.4 % (3483)	20.9 % (1160)	82.7 % (3483)	20.5 % (1160)	78.6 % (3483)	25.7 % (1160)
VP2 (2967, 988)	98.6 % (2967)	98.7 % (988)	85.1 % (2967)	17.3 % (988)	84.7 % (2976)	17.4 % (991)	81.5 % (2967)	22.1 % (988)
VP3 (2307, 768)	98.6 % (2307)	99.1 % (768)	87.2 % (2307)	14.6 % (768)	87.3 % (2307)	14.4 % (768)	79.0 % (2307)	25.5 % (768)
NSP1 (975, 324)	99.9 % (975)	99.7 % (324)	80.0 % (975)	75.4 % (324)	80.5 % (975)	76.2 % (324)	45.3 % (933)	25.4 % (310)
NSP2 (903, 300)	96.3 % (903)	99.0 % (300)	78.6 % (903)	74.1 % (300)	80.1 % (903)	76.4 % (300)	83.2 % (903)	80.4 % (300)
NSP3 (1098, 365)	99.8 % (1098)	100 % (365)	40.4 % (906)	19.7 % (301)	40.8 % (912)	14.2 % (303)	39.2 % (909)	27.4 % (302)
NSP4 (564, 187)	99.5 % (564)	100 % (187)	80.0 % (564)	83.0 % (187)	31.6 % (546)	11.2 % (181)	80.7 % (564)	79.3 % (187)
NSP5 (546, 181)	99.8 % (546)	100.0 % (181)	78.9 % (546)	77.5 % (181)	40.9 % (564)	11.0 % (187)	59.3 % (570)	49.5 % (189)

The turkey RVG strains from the healthy and infected hatcheries (MN-1 and MN-2, respectively) shared high amino acid sequences= identities in the remaining gene segments; 85.3 % in VP7, 97.9 % in VP4, 100 % in VP6, 99.8 % in VP1, 98.7 % in VP2, 99.1 % in VP3, 99.7 % in NSP1, 99.0 % in NSP2, 100 % in NSP3, 100 % in NSP4 and 100 % in NSP5 (Table 1). Since RVG was identified in both hatcheries, RVG could be a non-pathogenic virus in the turkey, and the clinical disease in MN-2 could be attributed to Salmonella infection. Conversely, RVG might be associated with clinical diseases due to strain variability in the VP7, since MN-1 and MN-2 were only 85 % identical. These hypotheses would have to be verified by further clinical experiments.

The nucleotide sequence identities of the VP7 and VP4 genes of the turkey strains to other RVG strains were 53.9–61.6 and 48.0–48.6 %, respectively, suggesting a novel G and P genotype in turkeys. However, at present it is difficult to classify RVG strains due to the limited number of complete RVG genome sequences. The turkey VP4 gene segment had relatively low (~37 %) amino acid identity to the other RVG strains (Table 1). Since VP4 is used for viral attachment, the low amino acid identity could be related to host restriction factors. Within the NSP1 gene, the pigeon strain HK18/2011 shared the lowest nucleotide identity (45.3 %) with the study strain MN-1 while the nucleotide identity was higher with the chicken strains (~80.0 %) (Table 1). The NSP3 protein can simultaneously interact with the eukaryotic translation initiation factor eIF4G and the 3′ end of the mRNA to regulate cellular protein synthesis in RVA infections [10, 33], and the NSP3 of the turkey RVG strains was 192 nucleotides longer than that of the other RVG strains. The biological implications of the greater length in the NSP3 protein of turkey RVG strains are unknown. The turkey NSP4 gene segment shared a 31.6 % nucleotide identity with that of the chicken strain Ger/03V2567 but had higher identity with the other RVG gene segments. In addition, the turkey RVG strains shared a 40.9 % nucleotide identity with Ger/03V2567 in NSP5, but also had a low nucleotide identity (59.3 %) with the pigeon strain (Table 1).

Phylogenetic analyses were implemented to evaluate the genetic relationship of our RVG strains with heterologous RVG chicken and pigeon using the maximum likelihood algorithm, with the GTR nucleotide substitution model (bootstrap analysis with 1000 replicates) (Fig. 2). In the VP6, VP1-VP3 and NSP1 phylogenetic trees, MN-1 and MN-2 share a common ancestor with the chicken RVG strains (Fig. 2c, d–f and g, respectively). In the VP7 and NSP5 phylogenetic trees, MN-1 and MN-2 share a common ancestor with the chicken RVG strain MRC-DPRU1679 (Fig. 2a and k, respectively). In the NSP3 phylogenetic tree, MN-1 and MN-2 have a closer genetic relationship with the pigeon RVG strain CHN/HK18/2011 than the chicken strains (Fig. 2i). The RVG phylogenetic trees indicate a wide gap in evolutionary information based on the long branch lengths in the different gene segments.

**Fig. 2.**
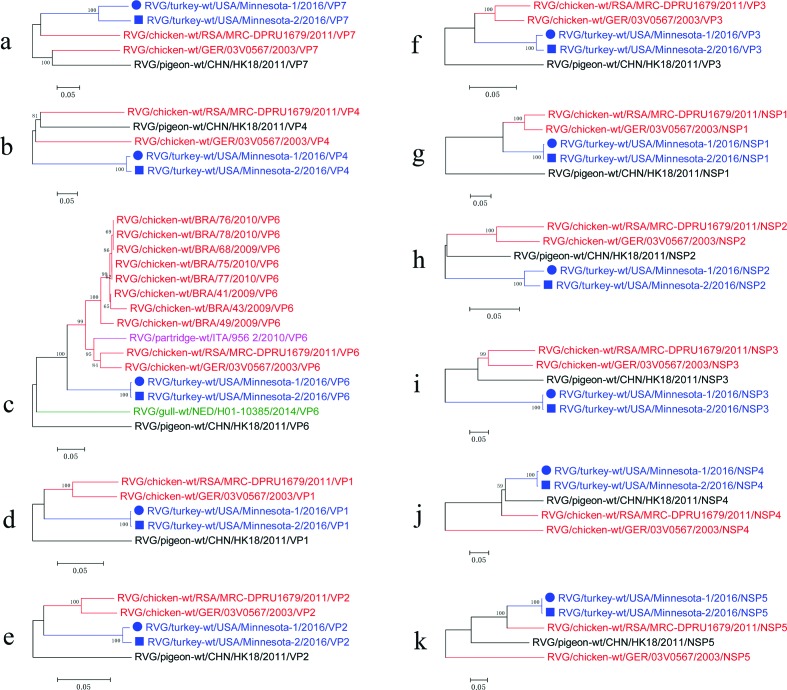
Maximum likelihood phylogenetic trees of different segments of the study strains with the cognate gene of the other RVG strains. Phylogenetic trees based on VP7 (a), VP4 (b), VP6 (c), VP1 (d), VP2 (e), VP3 (f), NSP1 (g), NSP2 (h), NSP3 (i), NSP4 (j) and NSP5 (k). Bootstrap values are represented at major nodes. Black solid circles and solid squares indicate the different segments of our RVG strains: RVG/turkey-wt/USA/Minnesota-1/2016 and RVG/turkey-wt/USA/Minnesota-2/2016, respectively. Scale bars indicate nucleotide substitutions per site.

Previously identified rotavirus motifs were present in both US turkey RVG strains. In the VP1 protein, the three conserved RNA-dependent RNA polymerase motifs (^558^AEKIILYTDVSQWDAS^573^, ^638^LRIRYHGVASGEKTTKIGNSFANVALI^664^ (different residues are highlighted by underlining) and ^683^MRVDGDDNVVT^693^) were identified, with the second motif representing a potential metal-binding motif [34]. Another conserved motif, ^456^ALYSLSN^462,^ was observed in the VP3 protein but its function remains unknown [35]. The motif ^744^KX[D/N]G^747^ in the VP3 protein is assumed to be associated with guanyltransferase activity [36]. In the RNA-binding domain of NSP2 protein, the conserved amino acid sequence ^229^HGXGHXRXV^237^ and histidine triad ^229^His-X-His-X-His-XX^235^ were observed; these facilitate the binding of nucleoside triphosphate [37].

In contrast to the avian RVA and RVD strains, knowledge of RVF and RVG is relatively scarce. RVG was first identified in broiler chicks with runting and stunting syndrome [22] while its role in these diseases remains to be clarified [25]. RVG has consistently been reported from South Africa, Germany and Italy [24, 26] in association with diarrhoea, growth depression and enteric diseases. While data on RVG are accumulating, the diversity of avian RVG, its infection status of in avian flocks and its role in avian disease require further investigation. RV strains were previously reported in 2–3-day-old poults [17, 38]. In a retrospective study on pathogen detection in cases of poult enteritis syndrome, the study concluded that Salmonella and enteric viruses, specifically RVs, occupy a niche in the intestines of 10day-old poults and may lead to poult enteritis syndrome at a very young age [38]. However, the RV species observed in these studies are unknown.

In conclusion, complete turkey RVG genomes of two strains were generated. Although RVG strains were identified in both a hatchery with no known health issues (MN-1) and one with high embryo/poult mortality (MN-2), the strains shared only 85.3 % nucleotide sequence identity and 88.4 % amino acid sequence identity in the VP7 gene segment. The turkey RVG strains shared some conserved motifs with other RVG strains. However, the RVG nucleotide and amino acid sequence identities ranged between 31.6–87.3 and 11.0–83.0 %, respectively, indicating high genetic diversity within the RVG species. The sections of high nucleotide identity noted in the VP6 and NSP3 gene segments suggest potential recombination events between turkey RVG and human RVB strains. The relationship between RVG and clinical disease cannot be confirmed at this time; clinical experiments are needed to characterize these newly identified turkey RVG strains. The diversity and classification of avian RVGs, the role of RVG in the avian disease and the inter-relation of human RVB and turkey RVG should be further investigated.
